# Characteristics of *Salmonella* Recovered From Stools of Children Enrolled in the Global Enteric Multicenter Study

**DOI:** 10.1093/cid/ciab051

**Published:** 2021-01-25

**Authors:** Irene N Kasumba, Caisey V Pulford, Blanca M Perez-Sepulveda, Sunil Sen, Nurulla Sayed, Jasnehta Permala-Booth, Sofie Livio, Darren Heavens, Ross Low, Neil Hall, Anna Roose, Helen Powell, Tamer Farag, Sandra Panchalingham, Lynette Berkeley, Dilruba Nasrin, William C Blackwelder, Yukun Wu, Boubou Tamboura, Doh Sanogo, Uma Onwuchekwa, Samba O Sow, John B Ochieng, Richard Omore, Joseph O Oundo, Robert F Breiman, Eric D Mintz, Ciara E O’Reilly, Martin Antonio, Debasish Saha, M Jahangir Hossain, Inacio Mandomando, Quique Bassat, Pedro L Alonso, T Ramamurthy, Dipika Sur, Shahida Qureshi, Anita K M Zaidi, Anowar Hossain, Abu S G Faruque, James P Nataro, Karen L Kotloff, Myron M Levine, Jay C D Hinton, Sharon M Tennant

**Affiliations:** 1 Center for Vaccine Development and Global Health; 2 Department of Medicine, University of Maryland School of Medicine, Baltimore, Maryland, USA; 3 Institute of Integrative Biology, University of Liverpool, Liverpool, United Kingdom; 4 Earlham Institute, Norwich Research Park, Norwich, United Kingdom; 5 School of Biological Sciences, University of East Anglia, Norwich, United Kingdom; 6 Centre pour le Developpement des Vaccins, Bamako, Mali; 7 Kenya Medical Research Institute/US Centers for Disease Control and Prevention, Kisumu, Kenya; 8 Division of Foodborne, Waterborne, and Environmental Diseases, Centers for Disease Control and Prevention, Atlanta, Georgia, USA; 9 Medical Research Council Unit, The Gambia at the London School of Hygiene and Tropical Medicine, Banjul, The Gambia; 10 Centro de Investigacao em Saude da Manhica (CISM), Maputo, Mozambique; 11 Catalan Institution for Research and Advanced Studies (ICREA), Pg. Lluís Companys 23, Barcelona, Spain; 12 Pediatric Infectious Diseases Unit, Pediatrics Department, Hospital Sant Joan de Déu (University of Barcelona), Barcelona, Spain; 13 Consorcio de Investigación Biomédica en Red de Epidemiología y Salud Pública (CIBERESP), Madrid, Spain; 14 ISGlobal, Hospital Clínic–Universitat de Barcelona, Barcelona, Spain; 15 Instituto Nacional de Saúde, Ministério de Saúde, Maputo, Mozambique; 16 National Institute of Cholera and Enteric Diseases, Kolkata, India; 17 Department of Pediatrics and Child Health, The Aga Khan University, Karachi, Pakistan; 18 International Centre for Diarrhoeal Disease Research, Mohakhali, Dhaka, Bangladesh; 19 Department of Pediatrics, University of Maryland School of Medicine, Baltimore, Maryland, USA

**Keywords:** moderate-to-severe-diarrhea (MSD), *Salmonella*, antibiotic susceptibility, serovars, gastroenteritis

## Abstract

**Background:**

The Global Enteric Multicenter Study (GEMS) determined the etiologic agents of moderate-to-severe diarrhea (MSD) in children under 5 years old in Africa and Asia. Here, we describe the prevalence and antimicrobial susceptibility of nontyphoidal *Salmonella* (NTS) serovars in GEMS and examine the phylogenetics of *Salmonella* Typhimurium ST313 isolates.

**Methods:**

*Salmonella* isolated from children with MSD or diarrhea-free controls were identified by classical clinical microbiology and serotyped using antisera and/or whole-genome sequence data. We evaluated antimicrobial susceptibility using the Kirby-Bauer disk-diffusion method. *Salmonella* Typhimurium sequence types were determined using multi-locus sequence typing, and whole-genome sequencing was performed to assess the phylogeny of ST313.

**Results:**

Of 370 *Salmonella-*positive individuals, 190 (51.4%) were MSD cases and 180 (48.6%) were diarrhea-free controls. The most frequent *Salmonella* serovars identified were *Salmonella* Typhimurium, serogroup O:8 (C_2_-C_3_), serogroup O:6,7 (C_1_), *Salmonella* Paratyphi B Java, and serogroup O:4 (B). The prevalence of NTS was low but similar across sites, regardless of age, and was similar among both cases and controls except in Kenya, where *Salmonella* Typhimurium was more commonly associated with cases than controls. Phylogenetic analysis showed that these *Salmonella* Typhimurium isolates, all ST313, were highly genetically related to isolates from controls. Generally, *Salmonella* isolates from Asia were resistant to ciprofloxacin and ceftriaxone, but African isolates were susceptible to these antibiotics.

**Conclusions:**

Our data confirm that NTS is prevalent, albeit at low levels, in Africa and South Asia. Our findings provide further evidence that multidrug-resistant *Salmonella* Typhimurium ST313 can be carried asymptomatically by humans in sub-Saharan Africa.

## Teaser

In agreement with prior studies, we show that nontyphoidal *Salmonella* is prevalent in stool of children <5 years, albeit at low levels, in Africa and South Asia and *Salmonella* Typhimurium ST313 can be carried asymptomatically by humans in sub-Saharan Africa.

*Salmonella enterica* subspecies *enterica* serovars Typhi (Typhi), Paratyphi A (Paratyphi A), and Paratyphi B *sensu stricto* (Paratyphi B) cause enteric fever, while nontyphoidal *Salmonella* (NTS) generally causes self-limited gastroenteritis in healthy individuals. However, in young infants, the elderly, and immunocompromised hosts, NTS can lead to bacteremia resulting in hospitalization and death [1]. In some resource-limited countries, NTS is a recognized etiologic agent of diarrhea [2–5] and an important risk factor for diarrhea-related morbidity and mortality in children [6]. In 2015, an estimated 37 410 children died as a result of NTS gastroenteritis, with a large burden of disease in Southeast Asia and South Asia [7]. Serovars Typhimurium and Enteritidis are the most common NTS isolated from cases of gastroenteritis worldwide. Despite the capacity to isolate *Salmonella* by stool culture, little is known about the prevalence of NTS serovars that cause gastroenteritis in Africa and South Asia.

Invasive NTS (iNTS) causes bacteremia in sub-Saharan Africa, occurring predominantly in infants, toddlers, as well as in malnourished or malaria-infected adults and/or those with human immunodeficiency virus (HIV) [8, 9]. Although the incidence of iNTS has declined in many sites across Africa [10], it is still one of the most common causes of bloodstream infections in African children [11]. Interestingly, unique clades of serovars Typhimurium and Enteritidis are associated with bacteremia in this region [8, 12, 13]. Most of the Typhimurium strains isolated from blood in sub-Saharan Africa belong to multi-locus sequence type (ST) 313 [14]. In contrast, the most common genotype isolated worldwide is ST19, which is generally associated with gastroenteritis [15] but has recently been reported as a primary cause of invasive infections in a study in Uganda [16]. Both ST19 and ST313 genotypes have been isolated from patients with either gastroenteritis or bacteremia in Kenya, although the number of diarrhea cases was low [17].

The use of antibiotics to treat uncomplicated NTS gastroenteritis in children is not recommended, except where progression to invasive disease is a risk [18, 19]. However, information about the antimicrobial susceptibility of NTS is useful as this knowledge contributes to our overall understanding of resistance markers that are circulating in specific geographic locations. In fact, NTS harboring antimicrobial resistance traits in the gastrointestinal tract could serve as a reservoir for iNTS [20]. Presently, countries with the highest burden of iNTS disease report 48–75% multidrug resistance to commonly used antibiotics, a major concern given that more-effective third-generation cephalosporins or fluoroquinolones may be less available or more costly in these settings [11, 21].

During 2007–2010, the Global Enteric Multicenter Study (GEMS) determined the etiologic agents of moderate-to-severe diarrhea (MSD) in children 0–59 months old living in The Gambia, Mali, Mozambique, Kenya, India, Bangladesh, and Pakistan [22]. This large, prospective, case-control study determined that NTS was significantly associated with MSD in infants (0–11 months) from the Bangladesh site and toddlers (12–23 months) and young children (24–59 months) from the Kenya site [22]. Here, we determined the prevalence of *Salmonella* serovars isolated in GEMS, evaluated antimicrobial susceptibility, identified Typhimurium sequence types, and examined the phylogenetic relatedness of Typhimurium ST313 isolates.

## METHODS

### GEMS Study Participants

The methods and main findings from GEMS have previously been described [22–24]. Briefly, GEMS participants were recruited from censused populations during 2007–2010 in The Gambia, Mali, Mozambique, Kenya, Bangladesh, India, and Pakistan. Study participants included children aged 0–59 months of age with MSD who presented to a sentinel health facility (see Supplementary Methods for additional details). Children were recruited into 0–11-, 12–23-, and 24–59-month age groups. For each child with MSD (case) enrolled, 1–3 children without diarrhea during the previous week (controls) were recruited. Scientific and ethics committees and institutional review boards of participating institutions in each country as well as the coordinating institution, University of Maryland, Baltimore, approved the study protocol prior to implementation. Informed consent was obtained in the local dialect from all participating caretakers before recruitment of their children into the study.

### Detection of *Salmonella* spp.

A panel of enteropathogens was identified from stool specimens, collected at the clinic from MSD cases or obtained at home by caregivers of children in the control group, as previously described [24]. *Salmonella* spp. were shipped to the Center for Vaccine Development and Global Health (CVD) at the University of Maryland School of Medicine for additional characterization.

### Characterization of *Salmonella* Serovars From Stools

At CVD, *Salmonella* spp. were agglutinated using polyvalent O and O1 antisera followed by serogroups O:2 (A), O:4 (B), O:6,7 and O:7 (C_1_), O:6,8 and O:8 (C_2_-C_3_), O:9 (D_1_), O:9,46 (D_2_), O:3,10 (E_1_), O:11 (F), and O:13 (G) antisera (Denka Seiken, Tokyo, Japan). Serovars Typhimurium, Typhi, Enteritidis, and Paratyphi B were fully serotyped (using O and H typing antisera) and additionally confirmed by polymerase chain reaction (PCR) [25, 26].

### Sequence Typing of Typhimurium Isolates

Sequence types were determined for all 87 Typhimurium isolates using multi-locus sequence typing (MLST) by PCR and sequencing and/or by examining whole-genome sequences. Sequence typing by MLST followed methodology described previously [15].

### Whole-Genome Sequencing and Phylogenetic Analysis

The majority of the *Salmonella* isolates (355 out of 370) were subjected to whole-genome sequencing. Following sequencing, 120 isolates were excluded from subsequent analyses as they did not meet the quality-control criteria. Details of sequencing and phylogenetic analyses are described in the Supplementary Methods.

### Antimicrobial Susceptibility Testing

The susceptibility of the 370 *Salmonella* isolates to chloramphenicol, ampicillin, ciprofloxacin, trimethoprim/sulfamethoxazole (TMP/SMX), gentamicin, and ceftriaxone was determined using the Kirby-Bauer disk-diffusion method and interpreted according to Clinical and Laboratory Standards Institute guidelines. Multidrug resistance was defined as resistance to ampicillin, chloramphenicol, and TMP/SMX. To assess whether the high resistance of NTS to antimicrobials was associated with antibiotic prescription rate, we determined the percentage of children with MSD (and *Salmonella* isolated in stools) who had been prescribed (but may or may not have been given) antimicrobial agents after visiting any of the sentinel health facilities that participated in GEMS.

### Statistical Analysis

To determine which individual *Salmonella* serovars were driving the association between *Salmonella* and MSD that was found in the original GEMS analyses [22], we used the same conditional logistic regression model as previously described [27]. Instead of including *Salmonella* species in the model we included variables for each *Salmonella* serogroup/serovar. The association of each serovar with MSD was adjusted for other co-pathogens. The rationale for this approach, generally, and in the unique context of GEMS, has been discussed previously [27]. Analyses were conducted using R version 3.3.2 (R Foundation for Statistical Computing). *P* values less than .05 were considered statistically significant.

## RESULTS

### Characteristics of Study Participants With Moderate-to-Severe Diarrhea

Of the cases with *Salmonella* identified, 86 (44%) were in the 0–11-month age group, 55 (29%) were in the 12–23-month age group, and 49 (26%) were in the 24–59-month age group (Table 1). Cases experienced severe signs of MSD. Approximately 20% of infants with *Salmonella* spp. detected had bloody diarrhea, while 100% of children aged 12–59 months old with *Salmonella* spp. produced watery diarrhea. Of note, a lower proportion of children with MSD who had *Salmonella* isolated tended to be female in all age groups.

**Table 1.** Characteristics of Children With Moderate-to-Severe Diarrhea and From Whom *Salmonella* Were Isolated

Clinical Signs and Symptoms	0–11 Months (n = 86)	12–23 Months (n = 55)	24–59 Months (n = 49)
Stool consistency			
Mucus	72.94	61.82	53.06
Pus	3.49	9.09	12.24
Bloody	24.42	12.73	0
Watery	75.58	87.27	100
Medical history			
Vomiting >3 times/day	40.70	40.0	48.98
Drank much less than usual	19.77	21.82	14.29
Very thirsty	59.3	67.27	83.33
Decreased activity or lethargy	36.05	54.55	53.06
Irritable or restless	45.35	61.82	55.10
Fever >38°C or parent perception	73.26	72.73	77.55
Physical examination			
Admitted to the hospital	17.44	18.18	22.45
Undernutrition	9.30	16.36	12.24
Loss of skin turgor	26.74	25.45	36.73
Dry mouth	54.65	74.55	81.63
Sunken eyes	65.12	85.45	87.76
Axillary temperature >38.3°C	18.60	21.82	26.53
Gender			
Female gender	37.21	45.45	40.82

Data are presented as percentages.

### Geographical Distribution and Prevalence of *Salmonella* Serovars

**Figure 1.**
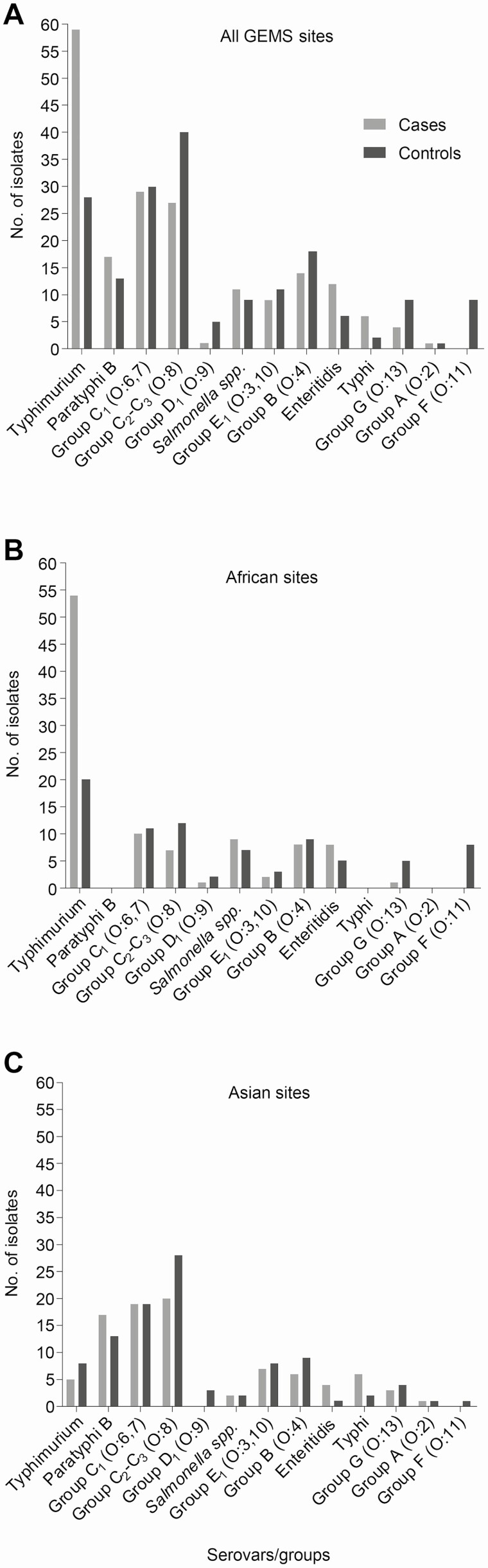
Distribution of *Salmonella* serogroups and serovars isolated from MSD cases and diarrhea-free asymptomatic controls. *Salmonella* spp. isolated from stools at (*A*) all 7 GEMS sites, (*B*) Africa, and (*C*) Asia. Abbreviations: GEMS, Global Enteric Multicenter Study; MSD, moderate-to-severe diarrhea.

The serovar distribution of the 370 *Salmonella* isolates (190 from cases and 180 from controls) collected from stools of study participants is shown in Figure 1. Of these, 361 were NTS. Additionally, we recovered 8 Typhi from Asia and 1 Paratyphi A isolate from Bangladesh. The most frequent NTS serovars identified were Typhimurium, serogroup O:8 (C_2_-C_3_), serogroup O:6,7 (C_1_), Paratyphi B Java, and serogroup O:4 (other than Typhimurium or Paratyphi B). Serovar Typhimurium predominated in Africa, whereas serogroup O:6,7 (C_1_) and O:8 (C_2_-C_3_) serovars were the most common in Asia.

### Prevalence of *Salmonella* Serovars by Site and Age Stratum

The prevalence of the most abundant serovars isolated in GEMS, as well as serovar Enteritidis (due to its importance in iNTS disease in Africa), is shown in Table 2. The individual serovars of isolates are listed in Supplementary Table 1. In general, we found that the rates of NTS isolation were low (≤5.3%) in both cases and controls regardless of age groups, although some site-to-site variation was apparent. At the Kenya site, serovar Typhimurium, the most prevalent serovar, was recovered in stools of MSD cases at a rate of 3.3% for infants, 3.7% for toddlers, and 4.3% for young children. In Bangladesh, Paratyphi B Java, the most prevalent serovar there, was recovered from 2.0% of infants with MSD. Serogroup O:8 (C_2_-C_3_) organisms were most prevalent in stools at the Pakistan site. The prevalence of NTS in cases and controls in The Gambia, Mali, Mozambique, and India was less than 1.5%.

**Table 2.** Prevalence of Nontyphoidal Salmonella at GEMS Sites Among Children With Moderate-to-Severe Diarrhea (Cases) and Controls

	Basse, The Gambia		Bamako, Mali		Manhica, Mozambique		Nyanza Province, Kenya		Kolkata, India		Mirzapur, Bangladesh		Karachi (Bin Qasim Town), Pakistan	
Prevalence of NTS	Cases	Controls	Cases	Controls	Cases	Controls	Cases	Controls	Cases	Controls	Cases	Controls	Cases	Controls
0–11 Months														
No. of participants	400	585	727	727	374	697	673	673	672	685	550	878	633	633
No. of NTS (%)	5 (1.3)	12 (2.1)	0	0	4 (0.8)	1 (0.1)	34 (5.1)	29 (4.3)	1 (0.1)	10 (1.5)	27 (4.9)	14 (1.6)	15 (2.4)	24 (3.8)
Typhimurium	0	0	0	0	0	0	22 (3.3)	9 (1.3)	0	2 (0.6)	1 (0.2)	0	1 (0.2)	1 (0.2)
Enteritidis	0	0	0	0	1 (0.3)	0	2 (0.3)	2 (0.3)	0	0	0	0	0	0
Paratyphi B Java	0	0	0	0	0	0	0	0	0	1 (0.5)	11 (2.0)	8 (0.9)	1 (0.2)	0
Serogroup O:4	0	0	0	0	0	1 (0.1)	2 (0.3)	5 (0.7)	0	1 (0.5)	1 (0.2)	0	2 (0.3)	2 (0.3)
Serogroup O:6,7	1 (0.3)	2 (0.3)	0	0	0	0	3 (0.4)	3 (0.4)	0	0	9 (1.6)	4 (0.5)	4 (0.6)	4 (0.6)
Serogroup O:8	0	1 (0.2)	0	0	1 (0.3)	0	2 (0.3)	4 (0.6)	1 (0.1)	1 (0.5)	5 (0.9)	1 (0.1)	7 (1.1)	13 (2.1)
Other serovars	4 (1.0)	9 (1.5)	0	0	2 (0.5)	0	3 (0.4)	6 (0.9)	0	5 (0.7)	0	1 (0.1)	0	4 (0.6)
12–23 Months														
No. of participants	455	639	682	695	195	391	410	621	588	598	476	761	399	676
No. of NTS (%)	6 (1.3)	8 (1.3)	1 (0.1)	0	1 (0.5)	0	24 (5.9)	21 (3.4)	1 (0.2)	2 (0.3)	7 (1.5)	9 (1.2)	14 (3.5)	19 (2.8)
Typhimurium	0	0	0	0	0	0	15 (3.7)	6 (1.0)	0	0	2 (0.4)	1 (0.1)	1 (0.3)	3 (0.4)
Enteritidis	0	0	0	0	0	0	4 (1.0)	2 (0.3)	0	0	2 (0.4)	0	1 (0.3)	1 (0.1)
Paratyphi B Java	0	0	0	0	0	0	0	0	0	0	3 (0.6)	2 (0.3)	0	0
Serogroup O:4	1 (0.2)	0	1 (0.1)	0	1 (0.5)	0	1 (0.2)	3 (0.5)	0	0	0	1 (0.1)	2 (0.5)	2 (0.3)
Serogroup O:6,7	2 (0.4)	4 (0.6)	0	0	0	0	0	2 (0.3)	1 (0.2)	1 (0.2)	0	1 (0.1)	0	4 (0.6)
Serogroup O:8	1 (0.2)	1 (0.2)	0	0	0	0	3 (0.7)	4 (0.6)	0	1 (0.2)	0	4 (0.5)	4 (1.0)	7 (1.0)
Other serovars	2 (0.4)	3 (0.5)	0	0	0	0	1 (0.2)	4 (0.6)	0	0	0	0	6 (1.5)	2 (0.3)
24–59 Months														
No. of participants	174	345	624	642	112	208	393	589	308	731	368	826	226	529
No. of NTS (%)	4 (2.3)	0	1 (0.2)	0	0	0	20 (5.1)	11 (1.9)	1 (1.0)	2 (0.3)	7 (2.2)	6 (0.7)	10 (5.8)	10 (2.3)
Typhimurium	0	0	0	0	0	0	17 (4.3)	5 (0.8)	0	0	0	0	0	1 (0.2)
Enteritidis	0	0	0	0	0	0	1 (0.3)	1 (0.2)	0	0	1 (0.3)	0	0	0
Paratyphi B Java	0	0	0	0	0	0	0	0	0	0	2 (0.5)	2 (0.2)	0	0
Serogroup O:4	0	0	1 (0.2)	0	0	0	1 (0.3)	0	0	0	0	0	1 (0.4)	3 (0.6)
Serogroup O:6,7	3 (1.7)	0	0	0	0	0	1 (0.3)	0	1 (0.3)	2 (0.3)	2 (0.5)	2 (0.2)	2 (0.9)	1 (0.2)
Serogroup O:8	0	0	0	0	0	0	0	2 (0.3)	0	0	0	0	3 (1.3)	1 (0.2)
Other serovars	1 (0.6)	0	0	0	0	0	0	3 (0.5)	0 (0.6)	0	2 (0.8)	2 (0.2)	4 (3.1)	4 (1.1)
Total participants (all ages)	1029	1569	2033	2064	681	1296	1476	1883	1568	2014	1394	2465	1258	1838
Total no. of NTS (all ages) (%)	15 (1.5)	20 (1.3)	2 (0.1)	0	5 (0.9)	1 (0.1)	78 (5.3)	61 (3.2)	3 (0.2)	14 (0.7)	41 (2.9)	29 (1.2)	39 (3.1)	53 (2.9)

Abbreviations: GEMS, Global Enteric Multicenter Study; NTS, nontyphoidal *Salmonella*.

### *Salmonella* Serovars Significantly Associated With Moderate-to-Severe Diarrhea

Previously, 3.2% and 3.7% of MSD episodes in toddlers (12–23 months) and children (24–59 months) at the Kenya site, respectively, and 4.6% of MSD episodes in infants at the Bangladesh site were shown to be attributable to *Salmonella* [22]. We determined the serovars driving the associations by using a conditional logistic regression model (Supplementary Table 2). In Bangladesh, serogroup O:6,7 (C_1_) (odds ratio [OR], 6.4; 95% confidence interval [CI]: 1.84–22.58), O:8 (C_2_-C_3_) (OR, 6.0; 95% CI: 1.28–28.33), and serovar Paratyphi B Java (OR, 4.8; 95% CI: 1.87–12.29) were significantly associated with MSD. In Kenya, the association was driven by serovar Typhimurium among children aged 12–23 months (OR, 4.3; 95% CI: 1.86–9.93) and 24–59 months (OR, 4.9; 95% CI: 2.09–11.64). All other serovars occurred in too few cases and controls to produce significant results.

### Antimicrobial Susceptibility at GEMS Sites

**Figure 2.**
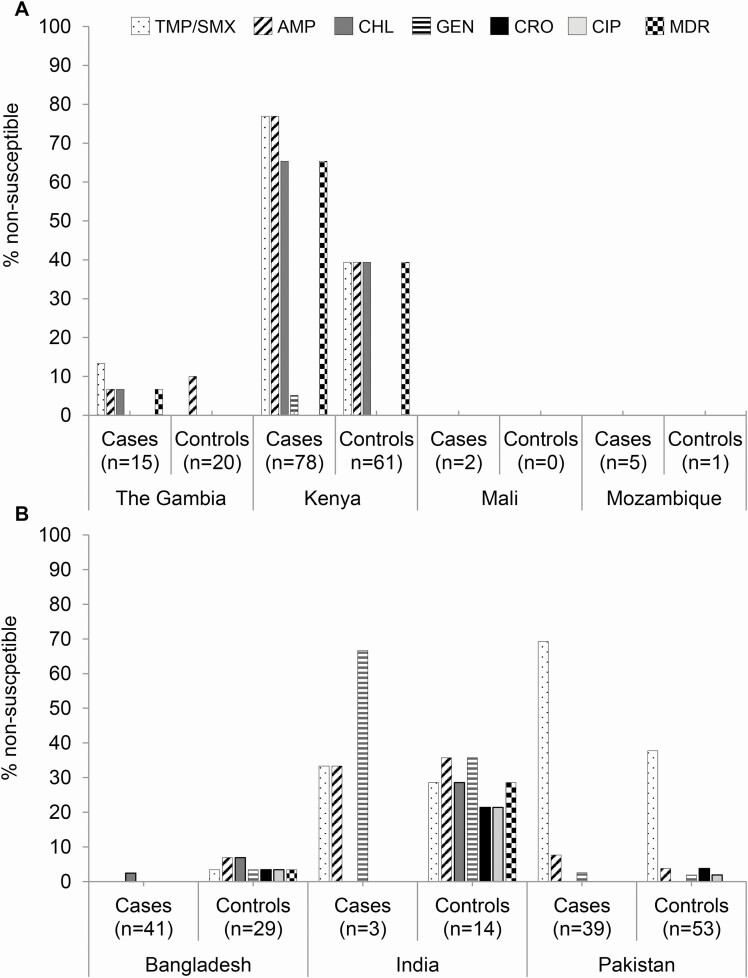
Percentage of NTS nonsusceptible to any of 6 commonly used antimicrobial agents. NTS isolated from (*A*) Africa and (*B*) Asia. Abbreviations: AMP, ampicillin; CHL, chloramphenicol; CIP, ciprofloxacin; CRO, ceftriaxone; GEN, gentamicin; MDR, multidrug resistant; NTS, nontyphoidal *Salmonella*; TMP/SMX, trimethoprim/sulfamethoxazole.

*Salmonella* isolates from the African and Asian sites differed in terms of their antimicrobial susceptibility (Figure 2). Isolates from Africa were susceptible to ciprofloxacin and ceftriaxone, whereas resistance to these antibiotics was observed among Asian NTS isolates. We observed 65.4% of NTS from MSD cases in Kenya to be multidrug resistant (MDR) to ampicillin, TMP/SMX, and chloramphenicol. However, isolation of nonsusceptible NTS was less frequent at the site in The Gambia; only 6.7% of NTS from cases showed an MDR phenotype (Figure 2A). In Asia, Indian isolates showed more resistance to the antibiotics tested than isolates from the other 2 Asian sites (Figure 2B). We observed antimicrobial susceptibility profiles among NTS from controls that were similar to cases at each site except for Kenya and India.

**Figure 3.**
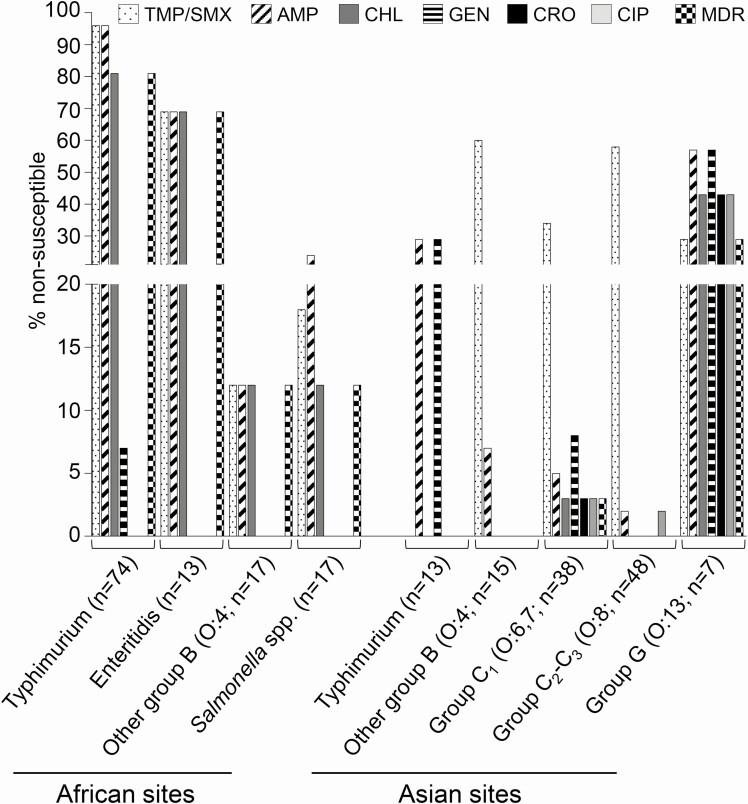
Percentage of NTS that were nonsusceptible to 6 antibiotics by serotype or serogroup. Only serotypes or serogroups that showed nonsusceptibility to antibiotics are shown. Abbreviations: AMP, ampicillin; CHL, chloramphenicol; CIP, ciprofloxacin; CRO, ceftriaxone; GEN, gentamicin; MDR, multidrug resistant; NTS, nontyphoidal *Salmonella*; TMP/SMX, trimethoprim/sulfamethoxazole.

Serovars Typhimurium and Enteritidis from Africa and serogroup O:6,7 (C_1_) and serogroup O:13 (G) isolates from Asia showed the highest percentage of antimicrobial resistance (Figure 3). All Enteritidis and Paratyphi B Java isolates recovered from GEMS stools from Asia were pan-susceptible to antimicrobial agents, while the 6 serogroup O:13 (G) isolates from Africa were pan-susceptible. Five of the 8 (62.5%) serovar Typhi from Asia (Pakistan and India) were MDR.

In general, most MSD cases with *Salmonella* had been prescribed or given an antibiotic (except in Pakistan) (Table 3). Trimethoprim/sulfamethoxazole was the most commonly prescribed antibiotic in Africa, whereas ciprofloxacin was the most common in Asia.

**Table 3.** Proportion of Children With Moderate-to-Severe Diarrhea and Positive for *Salmonella* Prescribed Any Antimicrobial Agents After Seeking Care at Sentinel Health Facilities That Participated in GEMS at Sites in Africa and Asia

	Africa	The Gambia	Mali	Mozambique	Kenya	Asia	India	Bangladesh	Pakistan
No. of cases^a^	100	15	2	5	78	90	5	42	43
No antibiotics prescribed/given, n (%)	23 (23.0)	4 (26.7)	0	0	19 (24.4)	36 (40.0)	0	1 (2.4)	35 (81.4)
Any antibiotics prescribed/given, n (%)	77 (77.0)	11 (73.3)	2 (100)	5 (100)	59 (75.6)	54 (60.0)	5 (100)	41 (97.6)	8 (18.6)
Antimicrobial agent, n (%)									
Ampicillin^b^	1 (1.0)	0	0	1 (20.0)	0	0	0	0	0
Chloramphenicol^b^	6 (6.0)	3 (20.0)	0	3 (60.0)	0	0	0	0	0
Ciprofloxacin/other fluoroquinolone^b^	5 (5.0)	1 (6.7)	0	0	4 (5.1)	43 (47.8)	4 (80.0)	31 (73.8)	8 (18.6)
Trimethoprim/sulfamethoxazole^b^	59 (59.0)	8 (53.3)	2 (100)	1 (20.0)	48 (61.5)	6 (6.7)	1 (20.0)	5 (11.9)	0
Gentamicin^b^	12 (12.0)	0	0	2 (40.0)	10 (12.8)	0	0	0	0
Amoxicillin	4 (4.0)	1 (6.7)	0	1 (20.0)	2 (2.6)	0	0	0	0
Azithromycin	0	0	0	0	0	4 (4.4)	0	4 (9.5)	0
Erythromycin	1 (1.0)	0	0	0	1 (1.3)	1 (1.1)	0	1 (2.4)	0
Penicillin	9 (9.0)	0	0	2 (40.0)	7 (9.0)	0	0	0	0
Selexid/pivmecillinam	0	0	0	0	0	0	0	0	0
Other macrolides	0	0	0	0	0	0	0	0	0

Abbreviation: GEMS, Global Enteric Multicenter Study.

^a^Total number of cases with moderate-to-severe diarrhea who were prescribed or given any antimicrobial agent at any participating sentinel health facility.

^b^Antimicrobial agents that were tested for susceptibility.

### Phylogenetic Analysis of *Salmonella* Typhimurium

Since Typhimurium was the most important cause of iNTS disease at several GEMS sites and was the most frequent serovar isolated from stools, a phylogenetic analysis was performed. Of 87 Typhimurium isolates, 74 (85.0%) were from Africa (Kenya), while 13 (14.9%) were from Asia (Pakistan, India, and Bangladesh). Table 4 shows the sequence types (ST) of these Typhimurium isolates listed by site of origin.

**Table 4.** Sequence Types of Typhimurium Isolates Identified in GEMS Stools Determined Using Multi-Locus Sequence Typing Polymerase Chain Reaction and/or Whole-Genome Sequencing

	Sequence Type		
Site	ST36	ST313	Total
Africa	0	74	74
Kenya	0	74	74
Asia	9	4	13
Bangladesh	4	0	4
Pakistan	3	4	7
India	2	0	2
All sites	9	78	87

Abbreviations: GEMS, Global Enteric Multicenter Study; ST, sequence type.

**Figure 4.**
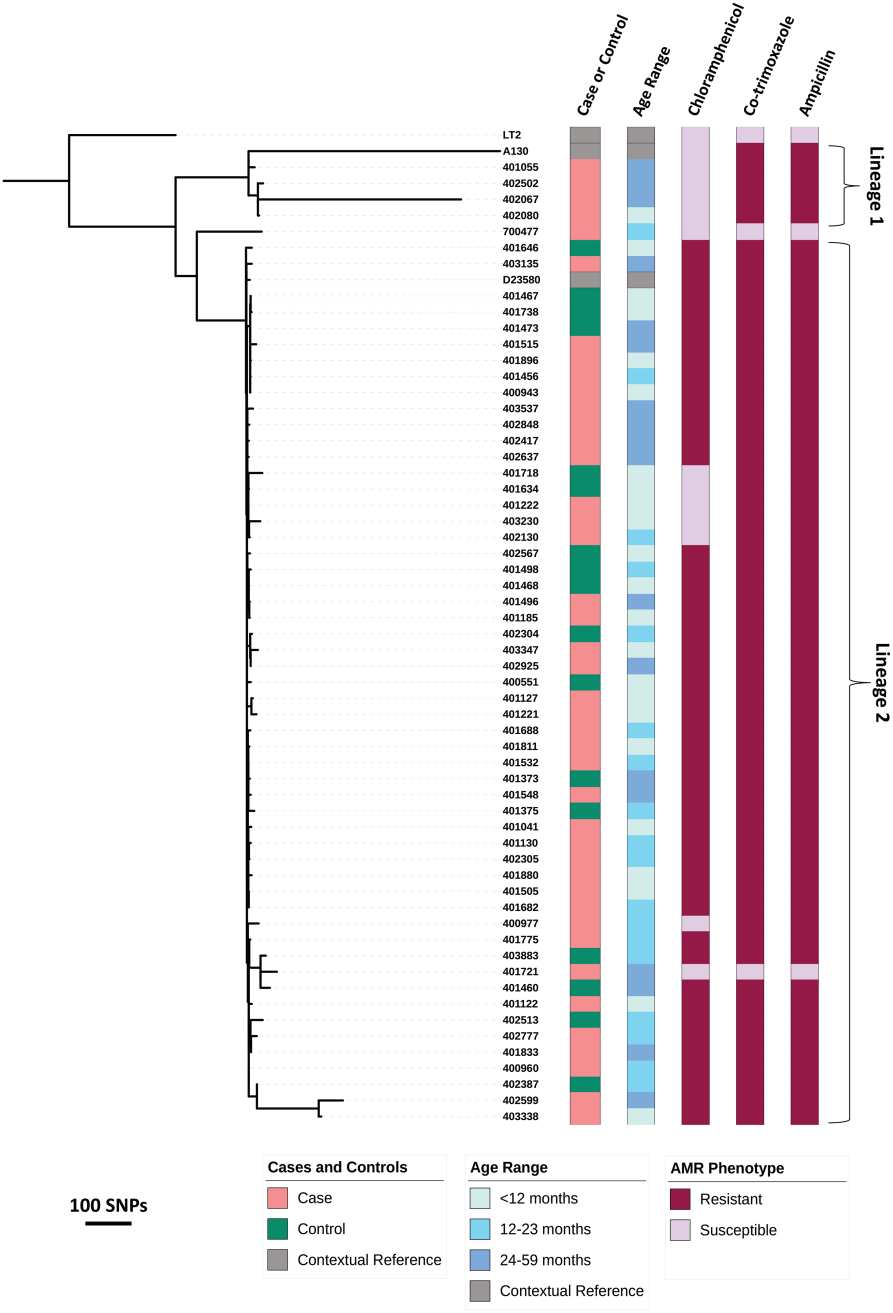
Genetic relationship between *Salmonella* Typhimurium ST313 isolated from MSD cases and diarrhea-free controls. The core genome maximum likelihood tree is shown for *Salmonella* Typhimurium ST313 isolated from the stool of cases and controls of children aged under 5 years in Kenya and Pakistan (1 isolate), which were collected as part of the GEMS. Scale bars in SNPs are shown beneath the phylogeny. Patient group, age range, and AMR data for the isolates are displayed using color strips created using Interactive Tree of Life (iTOL; Biobyte Solutions, Heidelberg, Germany) and are labeled and colored according to the in-laid key. Isolates that cluster with the lineage 1 or lineage 2 reference genomes are indicated. The tree is rooted using ST19. Abbreviations: AMR, antimicrobial resistance; GEMS, Global Enteric Multicenter Study; MSD, moderate-to-severe diarrhea; SNP, single nucleotide polymorphism; ST, sequence type.

A phylogeny was constructed using whole-genome sequences to determine the relationship between the Typhimurium ST313 isolates from MSD cases and controls (Figure 4). The African ST313 sequence type has been divided into the older lineage 1 isolates and the more recent lineage 2 [13, 28]. Here, 50 of 55 study isolates analyzed (90.9%) clustered with the ST313 lineage 2 reference genome D23580, 49 of 55 (89%) of which showed the typical MDR phenotype associated with lineage 2, namely resistance to chloramphenicol, co-trimoxazole, and ampicillin. The lineage 2 isolates from the MSD cases and diarrhea-free controls were closely related and could not be distinguished phylogenetically. A group of 5 isolates in stools of cases and controls, regardless of age, formed a small lineage 2 subcluster associated with susceptibility to chloramphenicol.

Four of the 55 isolates (7%) clustered with the ST313 lineage 1 reference genome A130, all of which were isolated from MSD cases in Kenya and were sensitive to chloramphenicol. Of note, the 1 study isolate (specimen 700477) that failed to cluster with lineage 1 or lineage 2 and demonstrated pan-susceptibility to antibiotics was isolated from a case of MSD from Pakistan.

## DISCUSSION

*Salmonella* isolates were detected in stools of children with MSD and from diarrhea-free community controls at each of the GEMS sites. A primary finding of our analysis was that, except for Typhimurium, the prevalence of most *Salmonella* serovars was similar in stools of cases and controls, regardless of age and across study sites. Because children enrolled as controls in GEMS only had to have been free of diarrhea for the previous 7 days, we could not rule out asymptomatic carriage or shedding of *Salmonella* among controls due to persistent excretion or convalescence [29]. Nontyphoidal *Salmonella* are reportedly excreted for longer periods in children than adults, lasting from several weeks to months [18, 30]. We found that NTS was as prevalent in cases as in controls, which suggests that NTS is endemic at the 7 GEMS sites [3, 31, 32].

In this study, we report the association of Typhimurium ST313 with acute diarrhea in Kenya using a conditional logistic regression model, showing that these bacteria cause diarrhea and are not just associated with invasive disease. This observation is supported by recent studies from Kenya, the Central African Republic, and Democratic Republic of Congo, which also detected Typhimurium ST313 in stool [17, 33, 34]. Typhimurium ST313 was identified in both MSD cases and controls, confirming that this important sequence type can be carried asymptomatically by humans. Phylogenetic analysis identified lineages 1 and 2, in accordance with previous findings [28]. We found that the same ST313 lineage (lineage 2) was prevalent in the stools of both MSD cases and controls. The fact that isolates from cases and controls are found in every part of the phylogeny suggests that the Typhimurium isolates that cause MSD are closely related to those associated with asymptomatic carriage; NTS carriage has been reported elsewhere in sub-Saharan Africa [34–36].

Several groups have attempted to identify the reservoir of iNTS isolates in Africa. Kariuki et al [20] were the first to suggest that these bacteria are not acquired zoonotically but are acquired by anthroponotic transmission. In this and other studies, NTS isolated from blood cultures of bacteremic index cases were highly similar to isolates from household contacts but different from NTS from animal or environmental sources taken from around the homes of index cases [20, 36]. Collectively, these prior studies suggest that the reservoir for Typhimurium ST313 is indeed humans. It remains possible that the lack of detection of *Salmonella* spp. from animals and the environment reflects difficulties in culture from these specimen types. However, if the inference from the above-mentioned studies is correct, our data would support these findings by showing that Typhimurium strains isolated from stools of cases and controls in GEMS are highly genetically related to isolates from blood.

When we examined the antimicrobial susceptibility of GEMS NTS isolates, we detected marked regional differences in resistance. We observed similar antimicrobial susceptibility patterns in stools of cases and asymptomatic controls at all GEMS sites except for Kenya and India. Our data suggest that antibiotic-resistant NTS are circulating in the GEMS communities. Nonsusceptible NTS strains could serve as a reservoir from which antibiotic-resistance determinants can spread horizontally to other microorganisms [37]. In Africa, the majority of Typhimurium and Enteritidis isolates were MDR, which is consistent with previous findings [20, 38]. Importantly, none of the isolates from GEMS African sites were resistant to ciprofloxacin or ceftriaxone, in contrast to isolates from Asia, suggesting a difference in utilization of these antibiotics. Five (of 8) Typhi from India and Pakistan were MDR but none were extensively drug resistant, as seen in the recent typhoid fever outbreak in Hyderabad, Pakistan [39].

Antibiotics are not recommended for the treatment of NTS gastroenteritis in pediatric patients due to the predisposition for extended excretion of bacteria and relapse of infection [18, 40, 41]. However, our data suggest that children with NTS disease are being prescribed antibiotics, which may have selected for resistant bacteria. We observed high prescription rates for ciprofloxacin and other fluoroquinolones in Asia and, not surprisingly, also high resistance of *Salmonella* to ciprofloxacin in Asia but not Africa (where ciprofloxacin was rarely prescribed). In contrast, we recorded high antibiotic prescription rates of TMP/SMX in Africa, which possibly led to the high resistance observed in Africa. TMP/SMX in combination with highly active antiretroviral therapy has been used routinely as prophylaxis for opportunistic infections in patients with HIV in Africa [42].

The low frequency of *Salmonella* in MSD cases from Mali, The Gambia, and Mozambique was somewhat unexpected given that these countries report high iNTS disease burdens [3, 31, 32]. However, the incidence of iNTS disease during GEMS (2007–2010) in these 3 countries decreased relative to earlier estimates, concomitant with a reduction in clinical malaria [3, 31, 32, 43]. Indeed, there is growing evidence to suggest that iNTS disease is correlated with clinical malaria and that efforts to control malaria have resulted in reduced iNTS disease incidence [9, 44]. A re-analysis of GEMS using quantitative molecular diagnostic methods showed higher attributable fractions for *Salmonella* in all age groups at all sites [45].

Our findings have 3 main implications: (1) the prevalence data could be used to refine incidence estimates for individual *Salmonella* serovars; (2) we report for the first time the association of Typhimurium ST313 with acute diarrhea, thereby showing that these bacteria are not just associated with invasive disease; and (3) our data demonstrate widespread asymptomatic carriage of ST313, a key cause of iNTS infections. Because we found that humans are carriers of MDR *Salmonella* strains that also cause iNTS [46], it is possible that these individuals serve as intermediaries in transmission and maintenance of these bacteria in the community.

## Supplementary Data

Supplementary materials are available at *Clinical Infectious Diseases* online. Consisting of data provided by the authors to benefit the reader, the posted materials are not copyedited and are the sole responsibility of the authors, so questions or comments should be addressed to the corresponding author.

ciab051_suppl_Supplementary_Table_S1

ciab051_suppl_Supplementary_Table_S2

ciab051_suppl_Supplementary_Data_S1

## Notes

***Financial support.*** This work was supported by grants from the Bill & Melinda Gates Foundation (grant numbers 38874, OPP1033572) to M. M. L. M. M. L. is supported in part by the Simon and Bessie Grollman Distinguished Professorship. This work was also supported by a Global Challenges Research Fund data and resources grant to the Earlham Institute (grant number BBS/OS/GC/000009D) and the Core Strategic Program of the Earlham Institute (grant number BB/CCG1720/1). Next-generation sequencing and library construction were delivered via the Biotechnology and Biological Sciences Research Council National Capability in Genomics and Single Cell at Earlham Institute (grant number BB/CCG1720/1), by members of the Genomics Pipelines Group. This project was also supported by the Wellcome Trust Senior Investigator Award (grant number 106914/Z/15/Z) to J. C. D. H. C. V. P. is supported by a Fee Bursary award and the John Lennon Memorial Scholarship awarded by the University of Liverpool.

***Potential conflicts of interest.*** S. M. T. and M. M. L. are co-inventors (covered by multiple patents) of a trivalent *Salmonella* (Enteritidis/Typhimurium/Typhi Vi) conjugate vaccine and live attenuated NTS vaccines. M. M. L. reports grants from the Bill & Melinda Gates Foundation during the conduct of the study; in addition, M. M. L. has a US patent 9 050 283, entitled “Broad Spectrum Vaccine Against Non-Typhoidal Salmonella,” issued 9 June 2015, licensed to Bharat Biotech International; a UK patent 2387417, entitled “Broad Spectrum Vaccine Against Non-Typhoidal Salmonella,” issued 11 May 2016, licensed to Bharat Biotech International; a France patent 2387417, entitled “Broad Spectrum Vaccine Against Non-Typhoidal Salmonella,” issued 11 May 2016, licensed to Bharat Biotech International; a Germany patent 2387417, entitled “Broad Spectrum Vaccine Against Non-Typhoidal Salmonella,” issued 11 May 2016, licensed to Bharat Biotech International; an India patent 312110, entitled “Broad Spectrum Vaccine Against Non-Typhoidal Salmonella,” issued 1 May 2019, licensed to Bharat Biotech International; a US patent 10 716 839, entitled “Compositions and Methods for Producing Bacterial Conjugate Vaccines,” issued 21 July 2020, licensed to Bharat Biotech International; and an India patent application 201717038528, entitled “Compositions and Methods for Producing Bacterial Conjugate Vaccines,” filed 30 October 2017, pending to Bharat Biotech International. S. M. T. reports grants from the Bill & Melinda Gates Foundation during the conduct of the study; in addition, S. M. T. has a US patent 9 050 283 licensed to Bharat Biotech for *Salmonella* vaccines, a US patent 9 011 871 licensed to Bharat Biotech for *Salmonella* vaccines, a patent for live attenuated nontransmissible *Salmonella* vaccine pending (application pending), and a patent for a broad-spectrum *Salmonella* vaccine pending (provisional patent application submitted). D. S. and A. S. G. F. report full-time employment with GSK Vaccines, after the conduct of the study. All other authors report no potential conflicts. All authors have submitted the ICMJE Form for Disclosure of Potential Conflicts of Interest. Conflicts that the editors consider relevant to the content of the manuscript have been disclosed.
