# Supplementary material for: Characteristics of Salmonella Recovered From Stools of Children Enrolled in the Global Enteric Multicenter Study
Source: Clin Infect Dis. 2021 Jan 25;73(4):631–41. doi: 10.1093/cid/ciab051 (PMC8366818; doi:10.1093/cid/ciab051)
Supplement: ciab051_suppl_Supplementary_Table_S2 [file ciab051_suppl_supplementary_table_s2.docx]

| GEMS site (age group) |  | AOR (95% CI) | p-value | Other pathogens included in the model |
| --- | --- | --- | --- | --- |
| Bangladesh (0-11 months) |  |  |  |  |
|  | Group B | 6.39 (0.10-420.91) | 0.3854 | ETEC any ST**, *Shigella*, *Aeromonas*, Rotavirus, *C. jejuni*/*C. Coli*, Adenoviru*s* 40/41, *Cryptosporidium*, and *E. histolytica* |
|  | Group C1 | 6.43 (1.84-22.58) | 0.0037 |  |
|  | Group C2-C3 | 6.01 (1.28-28.33) | 0.0233 |  |
|  | Group E1 | 3.77 (0.04-333.46) | 0.5622 |  |
|  | Paratyphi B | 4.79 (1.87-12.29) | 0.0011 |  |
|  | Typhimurium | 0.74 (0.01-67.56) | 0.8958 |  |
| Kenya (12-23 months) |  |  |  |  |
|  | Enteritidis | 1.56 (0.39-6.29) | 0.5287 | ETEC any ST, tEPEC, *Shigella*, Rotavirus, and *Cryptosporidium* |
|  | Group B | 0.91 (0.13-6.29) | 0.9200 |  |
|  | Group C1 | 0.24 (0.01-10.61) | 0.4619 |  |
|  | Group C2-C3 | 1.53 (0.44-5.35) | 0.5091 |  |
|  | Typhimurium | 4.29 (1.86-9.93) | 0.0007 |  |
|  | *Salmonella* spp.* | 1.10 (0.20-6.05) | 0.9125 |  |
| Kenya (24-59 months) |  |  |  |  |
|  | Enteritidis | 2.04 (0.28-15.20) | 0.4849 | ETEC any ST, *Shigella*, Rotavirus, and *Cryptosporidium* |
|  | Group B | 6.09 (0.18-207.76) | 0.3157 |  |
|  | Group C1 | 6.09 (0.18-207.76) | 0.3157 |  |
|  | Group C2-C3 | 0.15 (0.01-4.88) | 0.2871 |  |
|  | Group E1 | 0.78 (0.01-52.59) | 0.9081 |  |
|  | Group F | 0.28 (0.01-11.99) | 0.5036 |  |
|  | Typhimurium | 4.93 (2.09-11.64) | 0.0003 |  |
|  | | | | |

**Supplementary Table 2. Odds ratios for various *Salmonella* serovars identified in stools**

**Salmonella* isolates whose serovar could not be established beyond genus
**ETEC any ST – Enterotoxigenic *Escherichia coli* (ETEC) and any ETEC expressing heat-stable toxins (ST)
