# Supplementary material for: Characteristics of Salmonella Recovered From Stools of Children Enrolled in the Global Enteric Multicenter Study
Source: Clin Infect Dis. 2021 Jan 25;73(4):631–41. doi: 10.1093/cid/ciab051 (PMC8366818; doi:10.1093/cid/ciab051)
Supplement: ciab051_suppl_Supplementary_Data_S1 [file ciab051_suppl_supplementary_data_s1.docx]

**SUPPLEMENTARY METHODS**

**GEMS study participants**

Children were eligible for enrollment if they had a new acute diarrheal episode (≥3 loose stools/day; following 7 diarrhea-free days) with at least one of the following signs indicative of MSD: sunken eyes, loss of skin turgor, bloody diarrhea, administration of intravenous fluids, or admission to the hospital [1, 2]. Controls were matched on age, gender, and residence (lived in the same neighborhood as a child with MSD) within 2 weeks of the index case. Demographic and anthropometric measurements, medical histories and clinical symptoms were recorded upon study enrollment.

**Collection of stool specimens and identification of selected enteric pathogens**

Stool specimens, collected at the clinic from MSD cases or obtained at home by caregivers of children in the control group, were stored in refrigerated coolers. If antibiotics were to be administered before stool collection, a rectal swab was first obtained for culture; the remaining assays were performed on a whole stool sample collected within the ensuing ~12 hours. Stool samples and swabs placed in transport media arrived at diagnostic clinical laboratories within 18 hours of collection. A panel of enteropathogens was identified using classical clinical microbiology methods, reverse transcriptase (RT)-Polymerase Chain Reaction (PCR) for enteric viruses and enzyme-linked immunosorbent assay (ELISA) for viral and parasite pathogens.

**Identification of *Salmonella* by clinical microbiology methods**

If the agglutination with polyvalent O and O1 antisera was inconclusive, isolates were confirmed to be *Salmonella* spp. using Analytical Profile Index (API) strips (Biomerieux, Philadelphia, PA). Serovar nomenclature followed the White-Kauffman-Le Minor scheme (https://www.pasteur.fr/sites/default/files/veng_0.pdf). Typing of Phase 1 and 2 H antigens was performed using *Salmonella* grown on swarm media and H typing antiserum (Denka Seiken and Difco, BD Diagnostics, Sparks, MD). Serovar Paratyphi B isolates were tested for D-tartrate fermentation to differentiate between Paratyphi B *sensu stricto* (non-D-tartrate fermenter; causes paratyphoid fever) and Paratyphi B Java (D-tartrate fermenter; causes gastroenteritis and considered to be NTS).

**Sequence typing of Typhimurium isolates**

Briefly, freshly cultured bacterial colonies were emulsified in 300 µl ultra-pure water (Quality Biological Inc., Gaithersburg, MD) and boiled for 10 minutes, then spun at 9,000 *x g* for 5 minutes. Bacterial supernatant served as DNA template in the subsequent MLST amplification reactions. Seven predefined *Salmonella* alleles (*aroC, purE, dnaN, hemD, hisD, sucA and thrA*) were amplified using 10x Green Taq buffer, 10 mM deoxyribonucleotide triphosphate (dNTP), 2.5 U Green Taq DNA polymerase (GenScript, Piscataway, NJ), 20 µM forward and reverse primers and 5 µl of crude lysate DNA template in a 50 µl reaction. Products were amplified at 95°C for 3 minutes, followed by 30 cycles of 95°C for 30 seconds, 58°C for 40 seconds, and 72°C for 1 minute, followed by a 5-minute extension at 72°C. PCR products were electrophoresed on a 2% agarose gel to confirm amplification of each locus. PCR products were purified using the QIAquick PCR purification kit (Qiagen, Germantown, MD) and resuspended in 30 µl of deionized water. PCR products were sequenced in both directions using gene specific primers. Sequences were analyzed using Geneious (Biomatters Ltd, Auckland, New Zealand) software, and consensus sequences were used to query the *Salmonella* MLST database ([**http://mlst.warwick.ac.uk/mlst/dbs/Senterica**](http://mlst.warwick.ac.uk/mlst/dbs/Senterica)**)** to determine the Sequence Type (ST).

**Whole genome sequencing**

Isolates were sequenced as part of the 10,000 *Salmonella* Genomes project (<http://10k-salmonella-genomes.com>). 1 ng of genomic DNA was extracted (MagAttract kit, Qiagen) from all the isolates. Libraries were prepared with Nextera XT kit (Illumina, Inc.) and sequenced using paired-end 2 x 150 bp reads on the HiSeq 4000 System (Illumina, Inc.) resulting in a median genomic coverage of 30 X per sample. Paired-end reads underwent adapter trimming with Trimmomatic v0.36 [3] using ILLUMINACLIP in palindrome mode. Quality trimming was conducted using Seqtk v1.2-r94 (https://github.com/lh3/seqtk) with default error rate threshold of 0.05. Trimmed reads were assessed for quality using FastQC v0.11.5 (www.bioinformatics.babraham.ac.uk/projects/fastqc/) and MultiQC v1.0 (http://multiqc.info). Genomes were assembled using Unicycler v3.90 [4] and only the assemblies that met the *Salmonella* assembly quality criteria defined by Enterobase (<https://enterobase.warwick.ac.uk>) were used for downstream analysis. Specifically, the criteria were between 4 Mbp – 5.8 Mbp of assembled DNA sequence, N50 >20 Kb, <600 contigs, number of N’s per 100 Kb <3% and >70% sequence reads assigned to *Salmonella* spp. with Kraken v0.10.5-beta, and the 8Gb mini-Kraken dustmasked database [5]. All assemblies which passed quality control were annotated using Prokka v1.12 [6] and the serovars were predicted using SISTR [7]. Sequences were deposited in the European Nucleotide Archive (<https://www.ebi.ac.uk/ena>) (accession numbers shown in Supplementary Table 1).

**Phylogenetic analyses**

A core-genome alignment of 235 high-quality genomes was created using Roary v3.11.0 [8]. SNPs were extracted using SNP-sites v2.3.3 [8] and a core-genome SNP alignment was created, which consisted of 1,988 SNPs across 62 taxa, including contextual reference strains for *Salmonella* Typhimurium ST313 lineage 1 (A130) and lineage 2 (D23580) [9]. The accession number of the assembled D23580 genome is FN424405 (<https://www.ebi.ac.uk/ena>). The accession number for the raw sequence data for *Salmonella* Typhimurium A130 is ERA000075 (<https://www.ncbi.nlm.nih.gov/sra)>. The core-genome SNP alignment was used to infer a maximum likelihood phylogenetic tree using RAxML-NG v0.6.0 [10], using the general time-reversible GTR model with gamma distribution for site-specific variation and 100 bootstraps to assess support. The phylogeny was rooted using the ST19 reference strain LT2 (GenBank accession number AE006468; [11]) and was visualized using the Interactive Tree of Life v4.2 [12].

**REFERENCES**

1. Kotloff KL, Blackwelder WC, Nasrin D, et al. The Global Enteric Multicenter Study (GEMS) of diarrheal disease in infants and young children in developing countries: epidemiologic and clinical methods of the case/control study. Clin Infect Dis **2012**; 55 Suppl 4: S232-45.

2. Kotloff KL, Nataro JP, Blackwelder WC, et al. Burden and aetiology of diarrhoeal disease in infants and young children in developing countries (the Global Enteric Multicenter Study, GEMS): a prospective, case-control study. Lancet **2013**; 382(9888): 209-22.

3. Bolger AM, Lohse M, Usadel B. Trimmomatic: a flexible trimmer for Illumina sequence data. Bioinformatics **2014**; 30(15): 2114-20.

4. Wick RR, Judd LM, Gorrie CL, Holt KE. Unicycler: Resolving bacterial genome assemblies from short and long sequencing reads. PLoS Comput Biol **2017**; 13(6): e1005595.

5. Wood DE, Salzberg SL. Kraken: ultrafast metagenomic sequence classification using exact alignments. Genome Biol **2014**; 15(3): R46.

6. Seemann T. Prokka: rapid prokaryotic genome annotation. Bioinformatics **2014**; 30(14): 2068-9.

7. Yoshida CE, Kruczkiewicz P, Laing CR, et al. The *Salmonella In Silico* Typing Resource (SISTR): An Open Web-Accessible Tool for Rapidly Typing and Subtyping Draft *Salmonella* Genome Assemblies. PLoS One **2016**; 11(1): e0147101.

8. Page AJ, Taylor B, Delaney AJ, et al. SNP-sites: rapid efficient extraction of SNPs from multi-FASTA alignments. Microb Genom **2016**; 2(4): e000056.

9. Kingsley RA, Msefula CL, Thomson NR, et al. Epidemic multiple drug resistant *Salmonella* Typhimurium causing invasive disease in sub-Saharan Africa have a distinct genotype. Genome Res **2009**; 19(12): 2279-87.

10. Stamatakis A. RAxML version 8: a tool for phylogenetic analysis and post-analysis of large phylogenies. Bioinformatics **2014**; 30(9): 1312-3.

11. McClelland M, Sanderson KE, Spieth J, et al. Complete genome sequence of *Salmonella enterica* serovar Typhimurium LT2. Nature **2001**; 413(6858): 852-6.

12. Letunic I, Bork P. Interactive tree of life (iTOL) v3: an online tool for the display and annotation of phylogenetic and other trees. Nucleic Acids Res **2016**; 44(W1): W242-5.
